# Tumor-associated macrophages promote epithelial–mesenchymal transition and the cancer stem cell properties in triple-negative breast cancer through CCL2/AKT/β-catenin signaling

**DOI:** 10.1186/s12964-022-00888-2

**Published:** 2022-06-17

**Authors:** Xiangzhou Chen, Mingqiang Yang, Jiang Yin, Pan Li, Shanshan Zeng, Guopei Zheng, Zhimin He, Hao Liu, Qian Wang, Fan Zhang, Danyang Chen

**Affiliations:** 1 Guangzhou Key Laboratory of “Translational Medicine on Malignant Tumor Treatment”, Affiliated Cancer Hospital & Institute of Guangzhou Medical University, No.78 Hengzhigang Road, Guangzhou, 510095 Guangdong China; 2 Department of Pharmacy, Renmin Hospital of Wuhan University, No.99 Zhangzhidong Road, Wuhan, 430000 Hubei China

**Keywords:** Tumor-associated macrophages, Triple-negative breast cancer, Cancer stem cell, β-Catenin, CCL2

## Abstract

**Background:**

Triple-negative breast cancer (TNBC) is a highly aggressive subtype of breast cancer with poor prognosis and limited treatment. As a major component of the tumor microenvironment, tumor-associated macrophages (TAMs) play an important role in facilitating the aggressive behavior of TNBC. This study aimed to explore the novel mechanism of TAMs in the regulation of epithelial–mesenchymal transition (EMT) and cancer stem cell (CSC) properties in TNBC.

**Methods:**

Expression of the M2-like macrophage marker CD163 was evaluated by immunohistochemistry in human breast cancer tissues. The phenotype of M2 macrophages polarized from Tohoku-Hospital-Pediatrics-1 (THP1) cells was verified by flow cytometry. Transwell assays, wound healing assays, western blotting, flow cytometry, ELISA, quantitative polymerase chain reaction (qPCR), luciferase reporter gene assays, and immunofluorescence assays were conducted to investigate the mechanism by which TAMs regulate EMT and CSC properties in BT549 and HCC1937 cells.

**Results:**

Clinically, we observed a high infiltration of M2-like tumor-associated macrophages in TNBC tissues and confirmed that TAMs were associated with unfavorable prognosis in TNBC patients. Moreover, we found that conditioned medium from M2 macrophages (M2-CM) markedly promoted EMT and CSC properties in BT549 and HCC1937 cells. Mechanistically, we demonstrated that chemokine (C–C motif) ligand 2 (CCL2) secretion by TAMs activated Akt signaling, which in turn increased the expression and nuclear localization of β-catenin. Furthermore, β-catenin knockdown reversed TAM-induced EMT and CSC properties.

**Conclusions:**

This study provides a novel mechanism by which TAMs promote EMT and enhance CSC properties in TNBC via activation of CCL2/AKT/β-catenin signaling, which may offer new strategies for the diagnosis and treatment of TNBC.

**Video Abstract**

**Supplementary Information:**

The online version contains supplementary material available at 10.1186/s12964-022-00888-2.

## Background

Breast cancer is the most common cancer in women worldwide [1]. Triple-negative breast cancer (TNBC) is a subtype of breast cancer that is deficient in protein expression of the estrogen receptor (ER), progesterone receptor (PR), and human epidermal growth factor receptor 2 (HER2); constituting approximately 15–20% of breast cancers [2, 3]. TNBC is more aggressive and has a poorer prognosis than the other types of breast cancer. To date, no effective targeted therapy is available for TNBC [4]. Thus, it is imperative to understand the molecular mechanisms underlying the aggressive behavior of TNBC and provide a more effective approach for diagnosis and therapy.

TNBC cells characteristically behave as if they have acquired epithelial–mesenchymal transition (EMT) and breast cancer stem cell-like properties [5, 6]. EMT is a cellular process in which cells lose epithelial characteristics and acquire mesenchymal properties, and are involved in embryonic development, fibrosis, wound healing and cancer progression [7]. In cancers, EMT has been shown to trigger the dissociation of cancer cells from the primary site, which invades adjacent tissues and subsequently disseminates to distant organs. EMT is an important step occurring during progression of cancers to more aggressive stages [8]. Furthermore, EMT has been proposed to be one of the most important biological processes in the induction of stem cell properties [8–10]. This finding was initially observed in breast cancer [11]. Breast cancer stem cells (BCSCs), isolated from human breast cancers, are a subpopulation of cells with the unique ability to self-renew and re-establish a heterogeneous population of breast cancer cells [12, 13]. CD44, CD24 and ALDH1 are three major CSC markers in breast cancer. Evidence has shown that both CD44^+^/CD24^−^ and ALDH1-expressing BCSCs are enriched in TNBC and are thought to be responsible for the aggressive behavior of TNBC [14]. Growing evidence suggests an overlap between EMT and CSCs; additionally, crosslinking plays a critical role in various tumor functions, including tumor growth, malignant progression, metastasis and chemoresistance [10].

The complicated crosstalk between cancer cells and various components of the tumor microenvironment (TME) is also involved in malignant cancer progression, in addition to intrinsic alterations in cancer cells [15–17]. Macrophages are a major component of the TME in breast cancer and are often referred to as tumor-associated macrophages (TAMs) [18, 19]. Macrophages are recruited to the TME and polarized into classically activated (M1) and alternatively activated (M2) phenotypes [20]. TAMs, typically associated with an M2-like polarization state, create an immunosuppressive microenvironment by producing cytokines, chemokines and growth factors, thus favoring the malignant progression of breast cancer [21–23]. However, studies on the molecular mechanisms of the EMT pathway and CSC induction in response to TAMs are limited.

Deregulation of β-catenin signaling is an important event in cancer malignancies [24–26]. In various cancers, β-catenin protein is not only involved in invasion and metastasis through a cell adhesion role but also participates in the Wnt pathway [27]. β-Catenin activity is mediated by its interaction with T-cell factor/lymphoid enhancer factor (TCF/LEF) transcription factors. The target genes activated by β-catenin are factors associated with cell proliferation, survival and stem cell markers [28, 29].

In this study, we demonstrated a high infiltration of TAMs into TNBC tissues. TAMs, polarized into the M2-like type, promote EMT and CSC properties in TNBC cells. Moreover, we demonstrated that TAMs markedly increased β-catenin expression and activity via regulation of CCL2/AKT signaling. Thus, we propose that a new mechanism by which TAMs promote cancer progression in TNBC, may provide new strategies for the diagnosis and treatment of TNBC.

## Materials and methods

### Cell culture

The human breast cancer cell lines BT549 and HCC1937 were cultivated in Dulbecco’s modified Eagle’s medium (DMEM; Gibco, Grand Island, NY, USA) supplemented with 10% FBS (Gibco) and 1% penicillin/streptomycin (Gibco). The human monocytic leukemia cell line THP1 was cultured in RPMI 1640 medium (Gibco) with 10% FBS. All cell lines were incubated at 37 °C in a humidified atmosphere containing 5% CO_2_. The cell lines were authenticated utilizing short tandem repeat (STR) profiling every 6 months.

### Lentiviral transduction

Transfections were carried out using Lipofectamine 3000 reagent (Thermo Scientific) with Opti-MEM reduced serum medium (Thermo Scientific). For shRNA experiments, short hairpin sequences against the β-catenin gene and scrambled shRNA sequences were cloned into the lentiviral vector GV358 (GENECHEM, Shanghai, China). Using the packaging plasmids pHelper 1.0 and pHelper 2.0 (GENCHEM), lentivirus encoding β-catenin shRNA was generated and then infected into BT549 and HCC1937 cells. After 72 h of infection, cells were grown in medium containing 2 µg/mL puromycin (Sigma-Aldrich, St. Louis, MO, USA) to select cells with β-catenin knockdown.

### Immunohistochemistry

Primary tumor specimens were obtained from 105 patients diagnosed with breast cancer who underwent complete resection in the Affiliated Cancer Hospital of Guangzhou Medical University between 2008 and 2012. Follow-up information is obtained by reviewing the patients’ medical record. This study was approved by the Medical Ethics Committee of the Affiliated Cancer Hospital of Guangzhou Medical University and performed in accordance with the Declaration of Helsinki.

Immunohistochemistry for CD163 (anti-CD163 antibody, ab209664, Abcam) was performed using standard protocols. Briefly, paraffin-embedded sections of clinical breast cancer tissues were deparaffinized in xylene, rehydrated with graded alcohol and microwaved in 10 mM sodium citrate (pH 6.0) for 15 min. Hydrogen peroxide (0.3%) was applied to block endogenous peroxide activity. After incubation with 10% normal goat serum, the sections were incubated with primary antibodies in the dark overnight at 4 °C, followed by incubation with horseradish peroxidase (HRP)-conjugated antibody. The sections were visualized by a DAB visualization kit (Maixin. Bio, China), and then counterstained with hematoxylin.

### Transwell assay

Cells were seeded in Matrigel invasion chambers (Corning, New York, USA), and conditioned medium of macrophages was added into the lower chambers as a chemoattractant. After incubation for 48 h, noninvasive cells and Matrigel were removed with a cotton swab. The invasive cells were stained with crystal violet. Each lower side of the Transwell chambers was imaged and counted.

### Wound healing assay

Cells were seeded in 6-well plates and grown to confluence. After starving in serum-free medium for 24 h, the monolayer of cells was scraped with a sterile pipette. Cell migration to the wounded region was observed with a microscope, and images were acquired at 0, 24 and 48 h.

### Western blotting

Cells were lysed in RIPA buffer (Beyotime Biotechnology, China) containing protease inhibitor cocktail (Sigma). Lysates were centrifuged, separated on 10% SDS–polyacrylamide gels and transferred to a PVDF membrane (Millipore). Membranes were blocked with 5% skim milk powder in PBST, probed with primary antibodies overnight at 4 °C and finally incubated with appropriate horseradish peroxidase-coupled secondary antibodies. Visualization was performed using enhanced chemiluminescence (ECL) reagents (Thermo Scientific). The following antibodies were used in the western blotting assay including E-cadherin antibody (3195S, CST), N-cadherin antibody (13116S, CST), vimentin antibody (5741S, CST), Snail antibody (3879S, CST), β-Actin antibody (4970S, CST), SOX2 antibody (3579S, CST), OCT4 antibody (2750S, CST), Nanog antibody (4903S, CST), β-Catenin antibody (8480S, CST), p-β-Catenin (Ser552) antibody (5651S, CST), p-β-Catenin (Ser675) antibody (4176S, CST), Histone H3 antibody (26218S, CST), p-JNK antibody (4668S, CST), p-ERK2 antibody (4370S, CST) and p-Akt antibody (4060S, CST).

### Quantitative real time PCR

Total RNA was extracted with an E.Z,N.A.^®^ HP Total RNA Kit (Omega Biotek, Norcross, GA, USA). First-strand cDNA synthesis was performed using the PrimeScript^®^ RT Reagent Kit (TaKaRa, Shiga, Japan) according to standard procedures. Quantitative real-time PCR was performed on a CFX96 Real‐Time System (Bio‐Rad, Hercules, CA). The expression values were normalized to *GAPDH* as a housekeeping gene using the ddCt method. Primer sequences of human *GAPDH* gene were 5′-GTCTCCTCTGACTTCAACAGCG-3′ (forward) and 5′-ACCACCCTGTTGCTGTAGCCAA-3′ (reverse). Primer sequences of human *CCL2* gene were 5′-AGAATCACCAGCAGCAAGTGTCC-3′ (forward) and 5′-TCCTGAACCCACTTCTGCTTGG-3′ (reverse).

### Flow cytometry assay

For cell-surface analysis, cells were harvested by dissociation using 0.05% trypsin/EDTA. A total of 1 × 10^6^ cells were resuspended in 200 μL PBS with 1% FBS, incubated with antibodies at the recommended concentrations at 4 °C for 30 min, and then detected by flow cytometry (BD FACSCanto II, BD Biosciences, USA). Data were analyzed using FlowJo software. The antibodies used in flow cytometry assay were as follows: APC-conjugated CD163 antibody (333610, Biolegend), PE-conjugated CD206 antibody (321106, Biolegend), APC-conjugated CD44 antibody (559942, BD Biosciences), PE-conjugated CD24 antibody (555428, BD Biosciences).

### ALDEFLUOR assay

Cancer stem cell activity was measured using the ALDEFLUOR assay (Stemcell Technologies, Vancouver, BC, Canada). The assay was performed following the manufacturer’s instructions. Single cells were resuspended in 1 mL assay buffer. Five microliters of activated aldefluor reagent was added to the suspension in a test tube, mixed and immediately transferred into 0.5 mL of cell suspension to the control tube with 5 μL of DEAB reagent. Two tubes were incubated for 30 min at 37 °C, and cell suspensions were centrifuged and resuspended in 0.5 mL aldefluor assay buffer. The percentage of ALDH^+^ cells was measured using a BD FACSCanto II flow cytometer.

### Immunofluorescence assay

Cells were grown on 35-mm cell culture dishes with glass bottoms (NEST, Wuxi, China). Cells were fixed with 4% paraformaldehyde for 30 min. Fixed cells were washed and permeabilized with 0.1% Triton X-100 for 10 min. Cells were blocked with goat serum for 30 min and incubated with the anti-β-catenin antibody (1:100, 8480S, CST) overnight at 4 °C. After washing with PBS, the cells were subsequently incubated with Alexa Fluor^®^ 488-conjugated anti-rabbit IgG antibody (1:1000, ab150077, Abcam) for 1 h. The nuclei were stained with DAPI (62248, Thermo Scientific). Images were acquired using a Zeiss LSM710 confocal microscope (Zeiss, Germany) at 400× magnification.

### TOPFlash/FOPFlash reporter assay

The TCF Reporter Plasmid Kit (Merck KGaA, Darmstadt, Germany) was used to measure the response of breast cancer cells to conditioned medium from macrophages. A total of 1 μg TOPFlash or FOPFlash and Renilla luciferase reporters were co-transfected into BT549 or HCC1937 cells using Lipofectamine 3000 and incubated for 24 h. Then, the cells were treated with conditioned medium from macrophages for 24 h. Luciferase reporter activity was measured by the dual-luciferase assay (Promega) according to the manufacturer's protocol.

### ELISA

Cells were incubated for 48 h in 5 mL culture medium. Then, the supernatants were collected, centrifuged to pellet any detached cells and measured using a Human CCL2 Quantikine ELISA Kit (R&D Systems, Minneapolis, MN, USA). ELISA was performed according to the manufacturer’s instructions.

### Statistical analysis

Statistical analysis was performed using GraphPad Prism 7 (GraphPad, San Diego, CA, USA). For these experiments, ANOVA or *t* tests were used to evaluate differences between groups. Data are expressed as the mean ± standard error of the mean (SEM) from at least three independent experiments. The survival curves were obtained by the Kaplan–Meier method, and differences were compared by the log-rank test. *p* value less than 0.05 was considered statistically significant.

## Results

### High infiltration of tumor-associated macrophages in triple-negative breast cancer

**Fig. 1:**
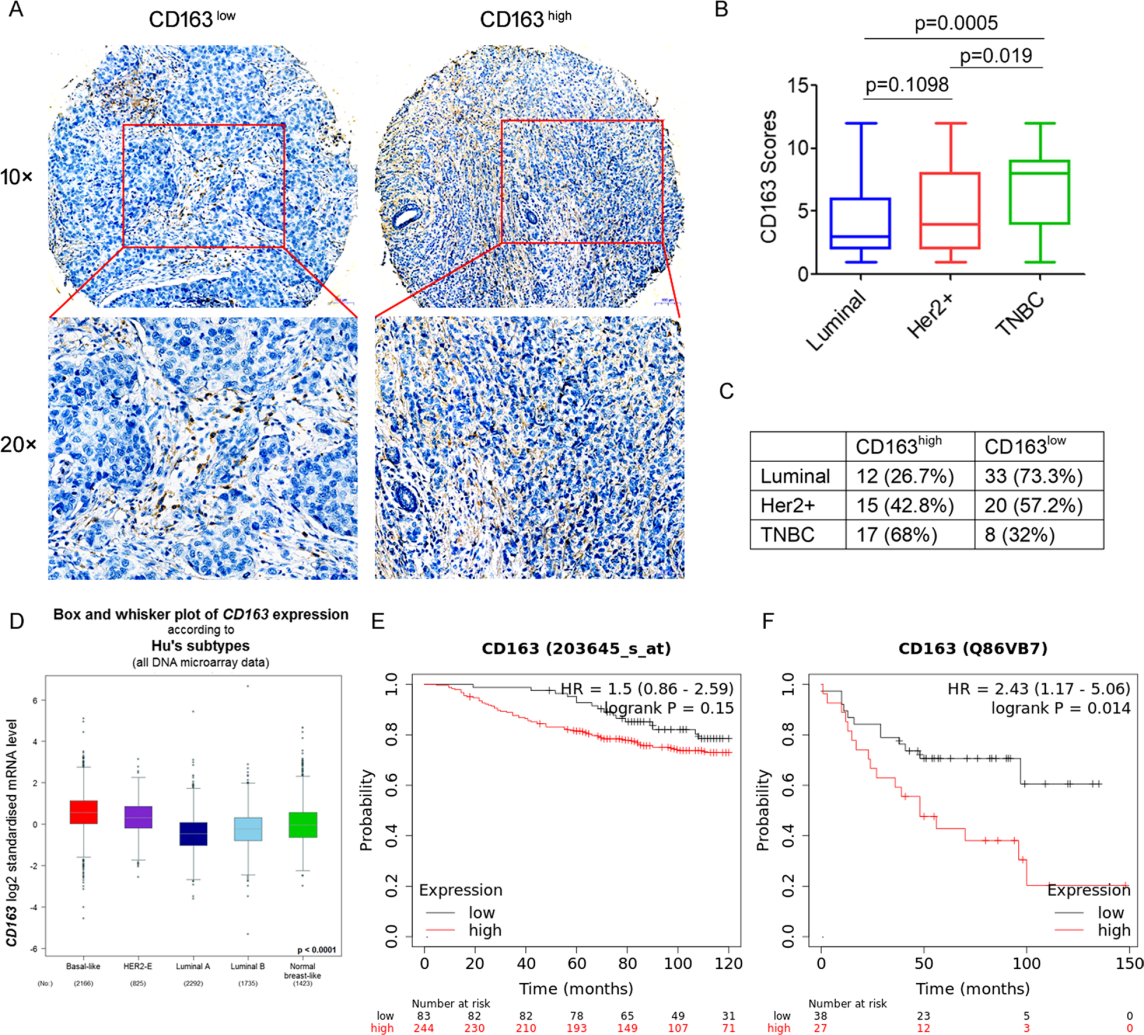
High infiltration of tumor-associated macrophages in triple-negative breast cancer. **A** Representative immunohistochemical staining for CD163 in tissue sections from human breast cancer patients. **B** CD163 expression scores in breast cancer tissue sections. **C** CD163 expression in different types of breast cancer tissues. **D** Box-and-whisker plots showing the expression level of CD163 among normal, Her2^+^, luminal A, luminal B and basal-like subtypes. **E**–**F** Kaplan–Meier plots for the survival probability of all breast cancer patients with high or low CD163 expression in mRNA (**E**) and protein (**F**) levels, respectively

First, we investigated the correlation between tumor-associated macrophages and human breast cancer. The expression of CD163, a highly specific marker of M2-like macrophages, was evaluated by immunohistochemistry in 105 human breast cancer tissue samples (Fig. 1A). We found that high expression of CD163 was detected in 43/105 (40.9%) breast cancer tissues, and high CD163 expression was found to be significantly correlated with lymph node metastasis (*p* = 0.012), but not with other clinicopathological parameters, such as age and TNM stage (Table 1). More importantly, we found that CD163 was differentially expressed in several types of breast cancer tissues, with a significantly higher proportion in triple-negative breast cancer tissues than in luminal and Her2-positive breast cancer tissues (Fig. 1B, C). Subsequently, the mRNA expression of CD163 was analyzed using publicly available TCGA datasets for breast cancer. As shown in Fig. 1D, the basal-like subtype expressed significantly higher levels of CD163 mRNA than luminal and Her2-positive breast cancer subtypes. Furthermore, according to the Kaplan‑Meier Plotter public database, high CD163 expression was associated with a substantially decreased survival probability in the basal-like TNBC population (*p* = 0.014, Fig. 1E, F). Collectively, these results suggest high infiltration of tumor-associated macrophages in triple-negative breast cancer.

**Table 1:** Correlation between CD163 expression and clinicopathological features of breast cancer

Characteristics	CD163^high^		CD163^low^		*p* value
	N	%	N	%	
	43	40.9%	62	59.1%	
Age					
< 50 (years)	19	44.2%	23	37.1%	0.537
> 50 (years)	24	55.8%	39	62.9%	
TNM stage					
I	11	25.6%	18	29%	0.118
II	12	27.9%	29	46.8%	
III–IV	20	46.5%	15	24.2%	
Lymph node metastasis					
Negative	13	30.2%	28	45.2%	0.012
Positive	30	69.8%	34	54.8%	

### TAMs promote epithelial–mesenchymal transition in TNBC cells

**Fig. 2:**
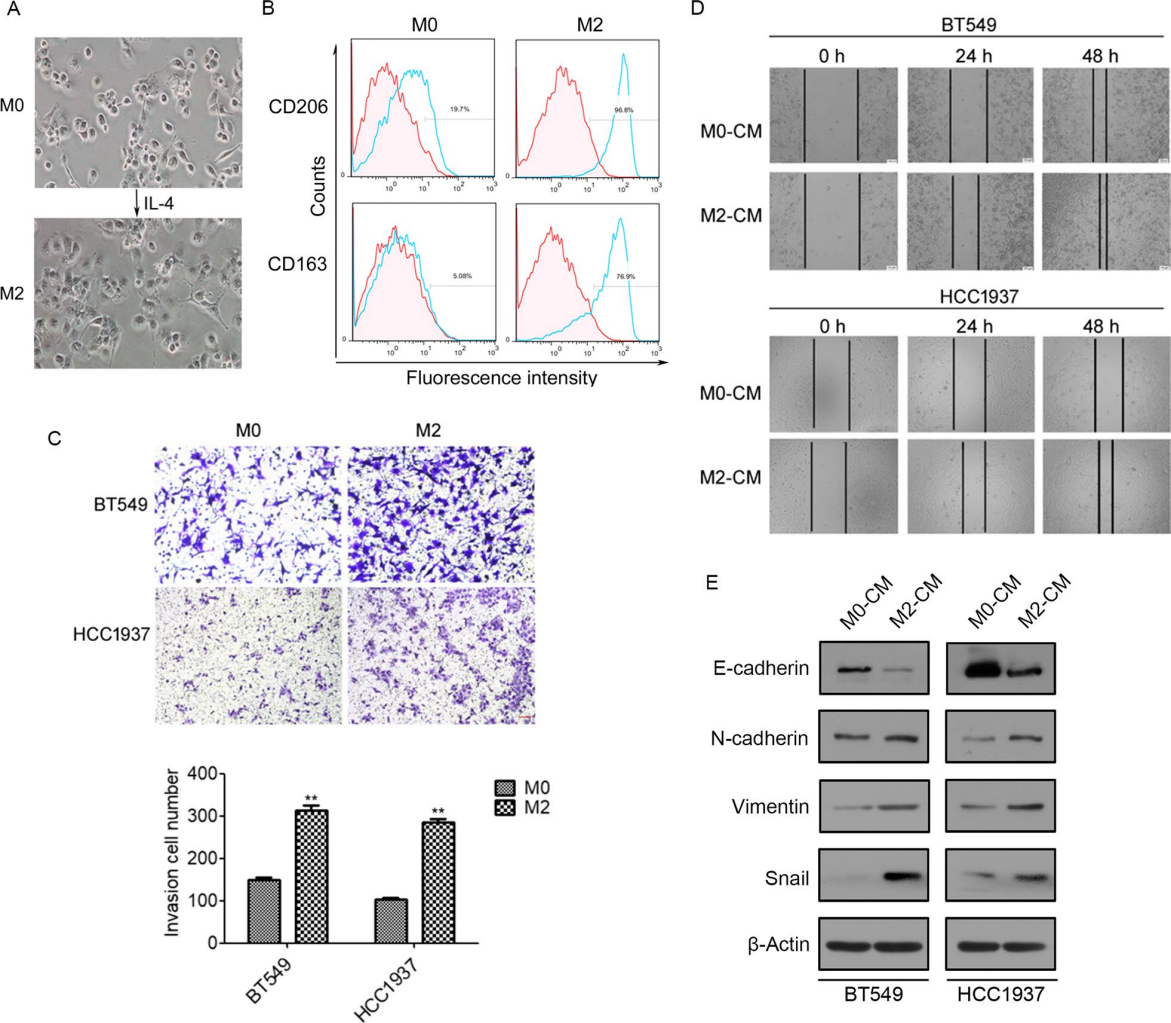
TAMs promote epithelial–mesenchymal transition in BT549 and HCC1937 cells. **A** Representative images of THP1 cells treated with IL-4 (20 ng/mL) for 48 h. **B** Flow cytometry analysis of the expression of CD206 and CD163 in M0 and M2 macrophages. **C** BT549 and HCC1937 cells were cultured with M2-CM or M0-CM, cell invasion were measured by transwell assay (up). Histogram showing the number of invasive BT549 and HCC1937 cells in transwell invasion assays (down). Scale bar = 100 μm. **D** The migratory ability of BT549 and HCC1937 cells treated with M0-CM or M2-CM was measured by wound healing assays. Scale bar = 100 μm. **E** BT549 and HCC1937 cells were cultured with M2-CM or M0-CM, the expression of E-cadherin, N-cadherin, vimentin, and Snail were measured using western blotting. N = 3. **p < 0.01

Previous studies suggested that tumor-associated macrophages (TAMs) exhibit an M2-like phenotype [16, 30, 31]. We produced polarized M2 macrophages from THP-1 cells by stimulation with IL-4 (20 ng/mL) for 48 h [32] (Fig. 2A). Flow cytometry analysis showed that the levels of M2 macrophage markers CD206 and CD163 were significantly increased by IL-4 treatment (Fig. 2B). Next, we investigated the function of M2 macrophages in the EMT in TNBC cells. Transwell assays showed that BT549 and HCC1937 cells cultured with the conditioned medium of M2 macrophages (M2-CM) exhibited significantly greater invasive capacity than those cultured with the conditioned medium of M0 macrophages (M0-CM) (Fig. 2C). Wound healing assays also showed that culture with M2-CM significantly increased the migration of BT549 and HCC1937 cells (Fig. 2D). We also investigated the effects of M2-CM on the expression of EMT markers. Protein expression of the mesenchymal markers N-cadherin and vimentin were dramatically increased, whereas the expression of epithelial marker E-cadherin was repressed after treatment with M2-CM in BT549 and HCC1937 cells (Fig. 2E). Meanwhile, the key transcription factor, Snail, was upregulated in BT549 and HCC1937 cells stimulated by M2-CM (Fig. 2E). Taken together, these findings suggest that M2 macrophages promote the EMT in TNBC cells.

### TAMs promote cancer stem cell properties in TNBC cells

**Fig. 3:**
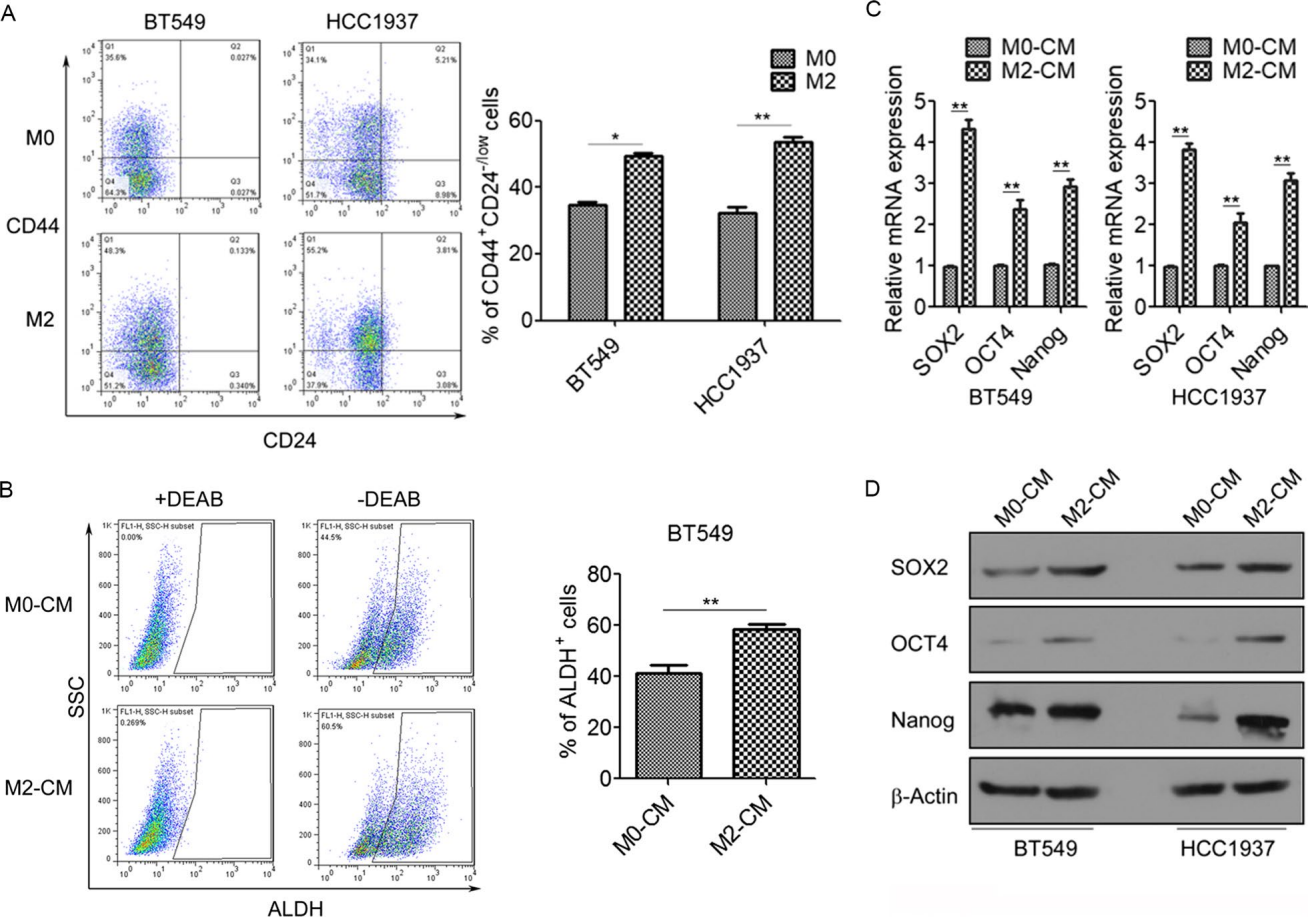
TAMs promote cancer stem cell properties in BT549 and HCC1937 cells. **A** Flow cytometry analysis for the CD44^+^/CD24^−^ cell populations in BT549 and HCC1937 cells cultured with M0-CM or M2-CM. **B** Flow cytometry analysis for the ALDH^+^ subpopulations in BT549 cells cultured with M0-CM or M2-CM. **C** qRT-PCR analysis of SOX2, OCT4, and Nanog mRNA levels in BT549 and HCC1937 cells cultured with M0-CM or M2-CM. **D** Western blotting analysis for SOX2, OCT4, and Nanog expression in BT549 and HCC1937 cells cultured with M0-CM or M2-CM. N = 3. *p < 0.05. **p < 0.01

EMT is associated with the generation and maintenance of cancer stem cells (CSCs), and we investigated the function of TAMs in the CSC properties of TNBC cells. Flow cytometry results revealed that treatment with M2-CM significantly elevated the CD44^+^/CD24^−^ subpopulations in BT549 and HCC1937 cells (Fig. 3A). ALDEFLUOR assays also showed that ALDH activity was markedly higher in M2-CM-treated BT549 cells than in M0-CM-treated BT549 cells (Fig. 3B). Moreover, M2-CM treatment significantly increased the mRNA and protein levels of CSC transcription factors SOX2, OCT4, and Nanog in BT549 and HCC1937 cells (Fig. 3C, D). These findings suggest that M2 macrophages promote CSC properties of TNBC cells.

### TAMs promote EMT and cancer stem cell properties via activation of β-catenin

**Fig. 4:**
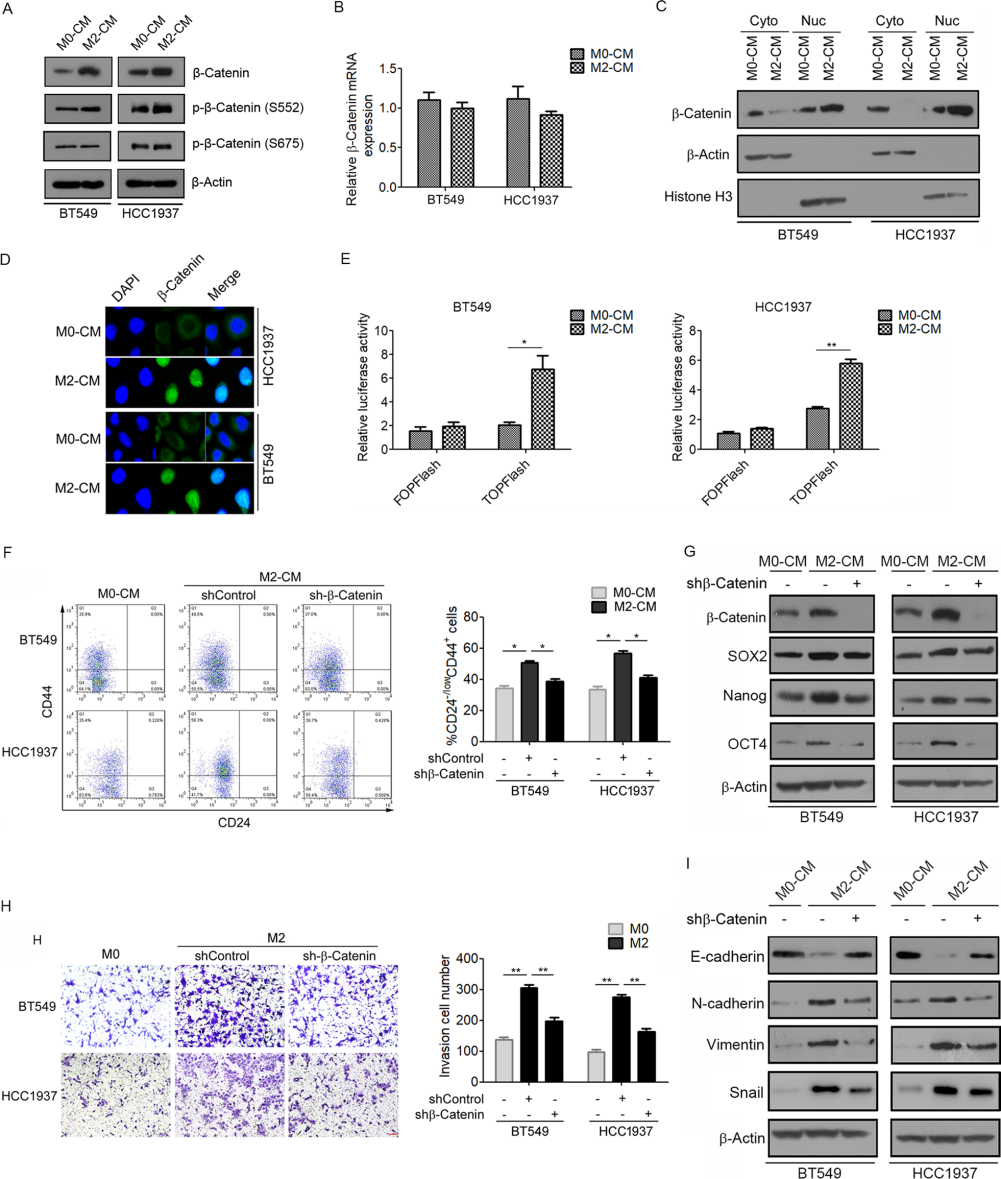
TAMs promote β-catenin expression and activity in BT549 and HCC1937 cells. **A** Western blotting analysis for protein levels of total β-catenin and phosphorylated β-catenin at Ser675 and Ser552 in BT549 and HCC1937 cells cultured with M0-CM or M2-CM. **B** qRT-PCR analysis of β-catenin mRNA levels in BT549 and HCC1937 cells cultured with M0-CM or M2-CM. **C** Western blotting showing nuclear and cytoplasmic protein levels of β-catenin in BT549 and HCC1937 cells. **D** Immunofluorescence staining of β-catenin in BT549 and HCC1937 cells cultured with M0-CM or M2-CM. **E** The relative luciferase activity of TOPFlash and FOPFlash in BT549 and HCC1937 cells cultured with M0-CM or M2-CM. **F**–**I** BT549 and HCC1937 cells were transfected with β-catenin shRNA followed by cultured M2-CM or M0-CM. **F** The CD44^+^/CD24^−^ cell populations were measured by flow cytometry. **G** The expression of β-catenin, SOX2, Nanog, and OCT4 were measured using western blotting. **H** Cell invasion were measured by transwell assay. **I** The expression of E-cadherin, N-cadherin, vimentin, and Snail was measured using western blotting. N = 3. *p < 0.05, **p < 0.01

Next, we investigated the mechanism by which M2 macrophages promote EMT and CSC properties in TNBC cells. Interestingly, M2-CM treatment significantly increased the protein expression of β-catenin in BT549 and HCC1937 cells (Fig. 4A). Meanwhile, M2-CM strongly stimulated the phosphorylation of β-catenin at Ser552, but not at Ser675 (Fig. 4A). However, the mRNA level of β-catenin was not changed by M2-CM treatment (Fig. 4B). Notably, we observed elevated nuclear localization of β-catenin in M2-CM-treated BT549 and HCC1937 cells, as detected using western blotting (Fig. 4C). These results were further verified by immunofluorescence (Fig. 4D). Subsequently, the role of M2-CM in β-catenin-mediated transcriptional activity was evaluated using TOPFlash/FOPFlash reporter assays. The results showed that M2-CM significantly increased β-catenin-driven transcriptional activity in BT549 and HCC1937 cells (Fig. 4E). These findings suggest that M2 macrophages promote β-catenin expression and activity in TNBC cells.

We further investigated whether TAMs promoted EMT and CSC properties in a β-catenin-dependent manner. We transfected BT549 and HCC1937 cells with β-catenin-specific shRNA and found that β-catenin knockdown markedly decreased the percentage of CD44^+^/CD24^−^ subpopulations (Fig. 4F) and the expression of the CSC transcription factors SOX2, OCT4, and Nanog (Fig. 4G) induced by M2-CM. Moreover, we examined the effects of β-catenin knockdown on the M2-CM-induced invasion of breast cancer cells. Transwell assays revealed that β-catenin knockdown significantly decreased the invasive ability of BT549 and HCC1937 cells induced by M2-CM (Fig. 4H). Consistently, the effects of M2-CM on the expression of E-cadherin, N-cadherin, vimentin, and Snail were reversed by β-catenin knockdown (Fig. 4I). These results suggest β-catenin activation mediates TAM-induced EMT and CSC properties in TNBC cells.

### TAMs activate β-catenin signaling by CCL2/AKT signaling

**Fig. 5:**
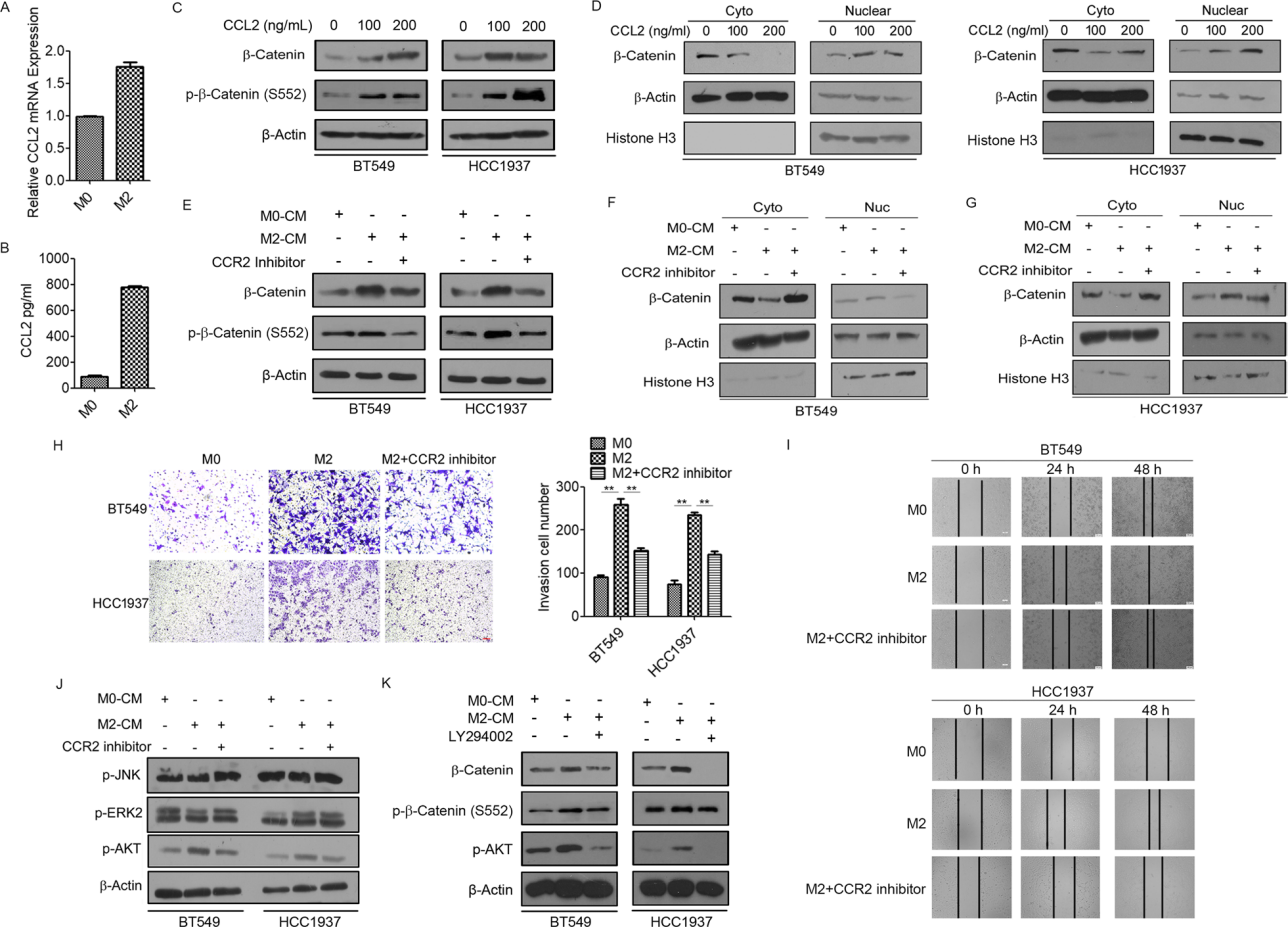
TAMs activate β-catenin signaling by CCL2/AKT signaling. **A** qRT-PCR analysis of CCL2 mRNA levels in M0 and M2 macrophages. **B** ELISA analysis of CCL2 secretion in M0 and M2 macrophages. **C** Western blotting analysis for total β-catenin and phospho-β-catenin (S552) levels in BT549 and HCC1937 cells induced by CCL2 at different concentrations. **D** Western blotting showing nuclear and cytoplasmic protein levels of total β-catenin in BT549 and HCC1937 cells induced by CCL2. **E** Western blotting analysis for total β-catenin and phospho-β-catenin (S552) levels in BT549 and HCC1937 cells cultured with M0-CM or M2-CM followed by treated with CCR2 inhibitor. **F**,** G** Western blotting showing nuclear and cytoplasmic protein levels of total β-catenin in BT549 and HCC1937 cells cultured with M0-CM or M2-CM followed by treated with CCR2 inhibitor. **H** Transwell assays showing the invasive ability of BT549 and HCC1937 cells cultured with M0-CM or M2-CM followed by treated with CCR2 inhibitor. Scale bar = 100 μm. **I** Wound healing assays showing the migratory ability of BT549 and HCC1937 cells cultured with M0-CM or M2-CM followed by treated with CCR2 inhibitor. Scale bar = 100 μm. **J** Western blotting analysis of the protein levels of p-JNK, p-ERK2 and p-AKT in BT549 and HCC1937 cells cultured with M0-CM or M2-CM followed by treated with CCR2 inhibitor. **K** Western blotting analysis for total β-catenin and phospho-β-catenin (S552) levels in BT549 and HCC1937 cells cultured with M0-CM or M2-CM followed by treated with AKT inhibitor LY294002. N = 3. *p < 0.05. **p < 0.01

Next, we explored the mechanism by which TAMs increased β-catenin activity in TNBC cells. Several studies have reported that chemokine (C–C motif) ligand 2 (CCL2), also known as monocyte chemotactic protein-1 (MCP-1), regulates CSC fate in the TME [33]. M2 macrophages secreted higher levels of CCL2 than M0 macrophages (Fig. 5A, B). We then determined the effect of CCL2 on β-catenin expression in BT549 and HCC1937 cells. As shown in Fig. 5C, D and Additional file 1: Fig. S1, CCL2 treatment significantly increased the total and nuclear levels of β-catenin as well as the phosphorylation status of β-catenin at Ser552. To confirm whether CCL2 secretion from M2 macrophages mediates the upregulation of β-catenin in TNBC cells, BT549 and HCC1937 cells were treated with a CCR2 inhibitor and cultured with M2-CM. Western blotting results revealed that treatment with M2-CM significantly increased β-catenin expression and the level of phospho-β-catenin at Ser552, whereas the CCR2 inhibitor decreased the expression and phosphorylation at Ser552 of β-catenin in M2-CM-treated BT549 and HCC1937 cells (Fig. 5E). Moreover, the CCR2 inhibitor dramatically reversed M2-CM-induced nuclear localization of β-catenin and phospho-β-catenin at Ser552 (Fig. 5F, G; Additional file 1: Fig. S1). To further validate these findings, we performed a Transwell invasion assay. As expected, the CCR2 inhibitor attenuated the invasive capability of M2-CM-treated BT549 and HCC1937 cells (Fig. 5H). Decreased cell migration was also observed in the wound-healing assay (Fig. 5I). Next, we focused on the signaling pathway activated by CCL2. M2-CM treatment significantly increased the levels of phosphorylated Akt in BT549 and HCC1937 cells, which were completely abrogated by the CCR2 inhibitor (Fig. 5J). Furthermore, LY294002, a PI3K/AKT inhibitor, effectively decreased the protein levels of total β-catenin and phospho-β-catenin at Ser552 in M2-CM-treated BT549 and HCC1937 cells (Fig. 5K). Collectively, these results suggest that TAMs activate β-catenin by CCL2/Akt signaling.

## Discussion

TNBC is a highly aggressive subtype of breast cancer with a poor prognosis and limited treatment. It has been established that the tumor microenvironment plays a vital role in the aggressive behaviors of cancer. As an important component of the TME, TAMs exhibit diverse impacts on cancer progression [34, 35]. Although the functions of TAMs have been characterized in various cancer types, their role in regulating the EMT pathway and CSC induction in TNBC has not been clearly explained. In this study, we identified a novel mechanism underlying the TAM-mediated EMT and stemness in TNBC.

CD163, a scavenger receptor, is regarded as a highly specific macrophage marker for M2 polarization and has been frequently used to identify TAMs in numerous studies [36]. Here, we focused on the role of CD163^+^ macrophages in TNBC. Increased infiltration of CD163^+^ macrophages was observed in the TNBC tissues. Moreover, CD163^+^ macrophage infiltration was correlated with poor survival in TNBC patients. Here, we used M2 macrophages polarized from THP1 cells to explore how TAMs affect EMT and stemness in TNBC cells.

EMT is a predominant event in the morphological transformation of cancer cells and contributes to their malignant biological properties, including invasion and metastasis. Recently, emerging evidence has shown that TAMs participate in EMT regulation in distinct cancer types. TAMs promote the EMT by secreting various cytokines, such as IL1β, IL-8, TNF-α, and TGF-β [16]. In the current study, we demonstrated that BT549 and HCC1937 cells undergo EMT after treatment with M2-CM (Fig. 2). Simultaneously, invasion was increased in these cell lines. We also found that M2-CM treatment enhanced the CSC properties of BT549 and HCC1937 cells. CSCs are a subset of cancer cells with stem-like features that are capable of self-renewal and differentiation [37]. It has been reported that CSC subpopulations strongly overlap with EMT phenotypes. In addition, cancer cells at primary sites that acquire EMT phenotypes promote the metastatic proliferation of cells with CSC phenotypes [38]. BCSCs, characterized as CD44^+^/CD24^−^ALDH^+^, display the highest invasive ability [39]. The link between CSCs and EMT is vital for cancer cell plasticity, in which cancer cells transform into malignant cells [40]. Although the interaction behaviors of TAMs and EMT have been extensively researched in recent years, the mechanism of CSCs modulation by TAMs in TNBC is poorly understood.

Interestingly, β-catenin mediates M2 macrophages-induced CSC properties in BT549 and HCC1937 cells. As shown in Fig. 4, M2-CM stimulation promoted the expression and nuclear localization of β-catenin as well as active β-catenin (S552) in TNBC cells. Aberrant β-catenin signaling has been implicated in multiple cancers, including breast cancer [41]. Previous studies have reported that β-catenin expression is correlated with poor clinical outcome in breast cancer [42, 43]. β-catenin plays an essential role as a member of the Wnt pathway, which regulates various cellular processes involved in cancer progression. Mechanistically, cytoplasmic β-catenin is abnormally increased and then translocates into the nucleus, where it interacts with the TCF/LEF family of transcription factors, leading to the activation of downstream target genes that are involved in proliferation, invasion, migration and stemness, such as c-myc, cyclin D1, EPCAM and CD44 [44, 45]. To further confirm the connection between β-catenin and cancer cell stemness, we assessed the effects of β-catenin knockdown on M2-CM-induced BT549 and HCC1937 cells. β-catenin silencing significantly reduced the percentage of CD44^+^/CD24^−^ CSCs and invasive capacity of BT549 and HCC1937 cells. Similar to our observations, Jang et al*.* reported that Wnt/β-catenin signaling regulates the self-renewal and migration of CSCs, thereby facilitating tumor growth and metastasis in breast cancer [45].

The molecular mechanism underlying TAM-induced β-catenin activation remains unclear. In the TME, the crosstalk between TAMs and cancer cells is dependent on many factors. In particular, cytokines and chemokines secreted by macrophages induce phenotypic and functional changes in both cell types and influence cancer development, including tumor growth, invasion, metastasis, and immune evasion [16]. The current study found that TAMs secretes CCL2 to enhance BCSC-like properties via the activation of β-catenin. CCL2 is a well-studied chemokine expressed by tumor cells, macrophages and stromal cells within the TME. CCL2 interacts with CCR2 to mediate TAM recruitment into the TME and macrophage polarization towards the protumor type [46, 47]. Several studies have reported a correlation between CCL2 expression and malignant events in cancer [31, 48, 49]. Moreover, TAM-induced activation of β-catenin signaling was remarkably suppressed by blocking the CCL2-CCR2 axis in BT549 and HCC1937 cells. These data suggest that CCL2 is required for TAM-induced β-catenin expression and its nuclear localization. We further demonstrated that TAM regulation of BCSC properties via CCL2 secretion was mainly dependent on the Akt pathway. Early studies have shown that the downstream targets of CCR2 include the PI3K/Akt signaling pathway [50, 51]. Similar to previous reports, we found that CCL2 activated Akt activity. Akt has been identified as an oncogene with serine/threonine kinase activity [52] and as an upstream regulator of β-catenin, stimulating β-catenin nuclear translocation and activation either directly through phosphorylation of β-catenin or indirectly through phosphorylation and inactivation of GSK3β [53, 54]. The phosphorylation of β-catenin at Ser552 by Akt can stabilize the protein, enhance its nuclear accumulation and increase its transcriptional activity [55, 56]. Our results revealed that CCL2 secreted by TAMs activated Akt signaling, which in turn promoted β-catenin phosphorylation at Ser552. Therefore, we propose that TAMs promote the EMT and enhance CSC properties via CCL2/Akt/β-catenin signaling in TNBC cells.

## Conclusions

In summary, our study highlights the role of TAMs in the regulation of EMT and stemness in TNBC. TAMs polarized to the M2-like type promoted EMT and CSC properties in BT549 and HCC1937 cells. More importantly, TAMs enhanced EMT and CSC properties via CCL2/Akt/β-catenin signaling. These findings provide new insights into the mechanism by which TAMs promote the aggressive behavior of TNBC, which may advance the development of macrophage-based strategies for TNBC diagnosis and treatment.

## Supplementary Information

**Additional file 1. **Additional results figures. Fig. S1 TAMs activate β-catenin signaling by CCL2/AKT signaling.** A** Western blotting showing nuclear and cytoplasmic protein levels of phospho-β-catenin (S552) in BT549 and HCC1937 cells induced by CCL2.** B** Western blotting showing nuclear and cytoplasmic protein levels of phospho-β-catenin (S552) in BT549 and HCC1937 cells cultured with M0-CM or M2-CM followed by treated with CCR2 inhibitor.

## Data Availability

The datasets obtained and analyzed for this study will be made available from the corresponding author in a reasonable request.

## Abbreviations

TAMs: Tumor-associated macrophages
EMT: Epithelial–mesenchymal transition
CSCs: Cancer stem cells
TNBC: Triple-negative breast cancer
TME: Tumor microenvironment
ALDH: Aldehyde dehydrogenase
CCL2: Chemokine (C–C motif) ligand 2
