# Supplementary material for: Tumor-associated macrophages promote epithelial–mesenchymal transition and the cancer stem cell properties in triple-negative breast cancer through CCL2/AKT/β-catenin signaling
Source: Cell Commun Signal. 2022 Jun 17;20:92. doi: 10.1186/s12964-022-00888-2 (PMC9205034; doi:10.1186/s12964-022-00888-2)
Supplement: Supplementary file 2 — Additional file 1. Additional results figures. Fig. S1 TAMs activate β-catenin signaling by CCL2/AKT signaling. A Western blotting showing nuclear and cytoplasmic protein levels of phospho-β-catenin (S552) in BT549 and HCC1937 cells induced by CCL2. B Western blotting showing nuclear and cytoplasmic protein levels of phospho-β-catenin (S552) in BT549 and HCC1937 cells cultured with M0-CM or M2-CM followed by treated with CCR2 inhibitor. [file 12964_2022_888_MOESM2_ESM.docx]

**
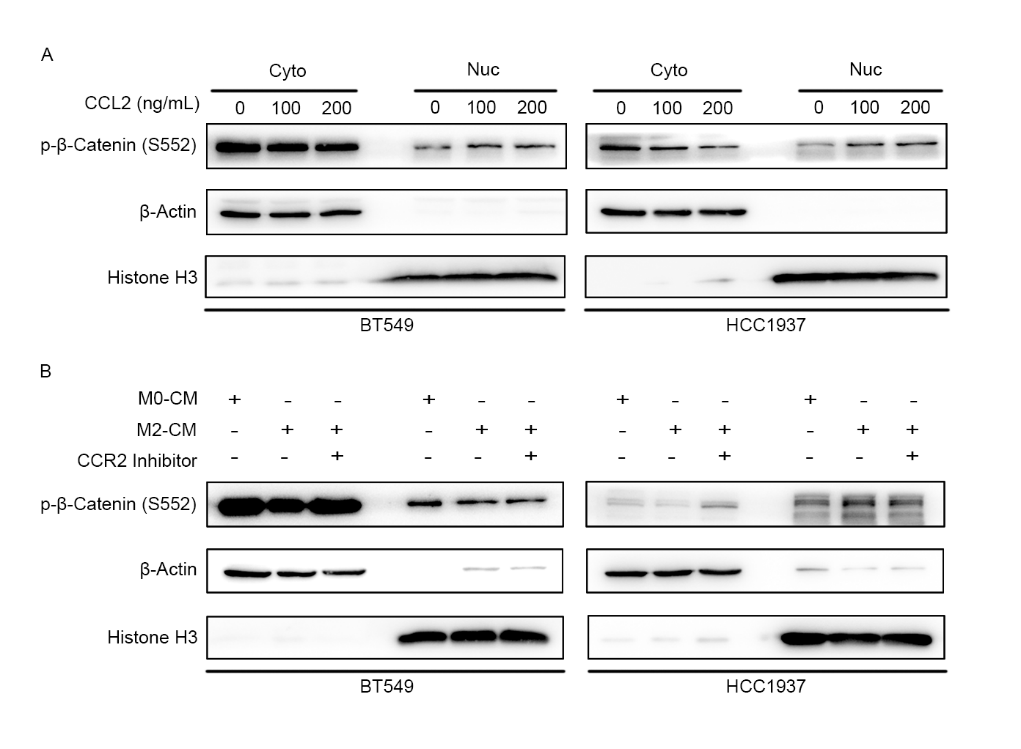
**

**Fig. S1 A** Western blotting showing nuclear and cytoplasmic protein levels of phospho-β-catenin (S552) in BT549 and HCC1937 cells induced by CCL2. **B** Western blotting showing nuclear and cytoplasmic protein levels of phospho-β-catenin (S552) in BT549 and HCC1937 cells cultured with M0-CM or M2-CM followed by treated with CCR2 inhibitor.
